# The role of GAPDH in the selective toxicity of CNP in melanoma cells

**DOI:** 10.1371/journal.pone.0300718

**Published:** 2024-03-21

**Authors:** Claudia von Montfort, Elif Aplak, Lara Ebbert, Chantal-Kristin Wenzel, Niklas P. Klahm, Wilhelm Stahl, Peter Brenneisen

**Affiliations:** Institute of Biochemistry and Molecular Biology I, Medical Faculty, Heinrich-Heine University, Düsseldorf, Germany; Rutgers University, UNITED STATES

## Abstract

**Background:**

Malignant melanoma is the most aggressive form of skin cancer with a rather poor prognosis. Standard chemotherapy often results in severe side effects on normal (healthy) cells finally being difficult to tolerate for the patients. Shown by us earlier, cerium oxide nanoparticles (CNP, nanoceria) selectively killed A375 melanoma cells while not being cytotoxic at identical concentrations on non-cancerous cells. In conclusion, the redox-active CNP exhibited both prooxidative as well as antioxidative properties. In that context, CNP induced mitochondrial dysfunction in the studied melanoma cells via generation of reactive oxygene species (primarily hydrogen peroxide (H_2_O_2_)), but that does not account for 100% of the toxicity.

**Aim:**

Cancer cells often show an increased glycolytic rate (Warburg effect), therefore we focused on CNP mediated changes of the glucose metabolism.

**Results:**

It has been shown before that glyceraldehyde 3-phosphate dehydrogenase (GAPDH) activity is regulated via oxidation of a cysteine in the active center of the enzyme with a subsequent loss of activity. Upon CNP treatment, formation of cellular lactate and GAPDH activity were significantly lowered. The treatment of melanoma cells and melanocytes with the GAPDH inhibitor heptelidic acid (HA) decreased viability to a much higher extent in the cancer cells than in the studied normal (healthy) cells, highlighting and supporting the important role of GAPDH in cancer cells.

**Conclusion:**

We identified glyceraldehyde 3-phosphate dehydrogenase (GAPDH) as a target protein for CNP mediated thiol oxidation.

## 1. Introduction

Malignant melanoma is based on genetic and epigenetic changes in melanocytes, being the pigment-producing cells of the skin [1]. The incidence of melanoma increased over the past decades, and it is now one of the most common cancers in young adults [2]. Malignant melanoma is still associated with a poor prognosis and a high mortality. As the standard chemotherapeutical therapy such as chemotherapy and/or radiation therapy has a lot of adverse effects, research on alternative therapies is highly needed.

Recently, nanoparticles were applied for various biomedical applications, including drug delivery, imaging, and cancer therapy [3]. Especially in context of cancer therapy, cerium oxide nanoparticles (CNP) are promising as their mixed valance state of Ce^3+^ and Ce^4+^ allows them to modulate the redox status of the cell [4]. Depending on the surrounding pH, size and exposure route, CNP either show superoxide dismutase (SOD) mimetic activity or catalase mimetic activity [5, 6]. Due to their SOD mimetic activity, treatment of cells with CNP increases the amount of intracellular hydrogen peroxide (H_2_O_2_) [5, 7]. As tumor cells already show a higher reactive oxygen species (ROS) load than normal cells [8], CNP treatment leads to a ROS level above a threshold value the cancerous cell can no longer cope with. Our previous data suggest that high levels of H_2_O_2_ result in mitochondrial dysfunction and cell death [7].

Several cancer cells preferentially use glucose for energy production, even in the presence of oxygen [9, 10]. Also, some malignant melanoma cells, among them A375, have been shown to have a high glycolytic activity compared to normal human fibroblasts [11]. This effect is described as the Warburg effect, also known as aerobic glycolysis [9, 12]. Normal cells typically rely on oxidative phosphorylation in the mitochondria to produce energy, which is a more efficient process than glycolysis. However, cancer cells often have a high rate of glucose uptake and convert glucose to lactate through glycolysis, even in the presence of sufficient oxygen, resulting in a less efficient energy production process. This metabolic switch is now thought to support the high energy demand of rapidly dividing cancer cells and to provide necessary intermediates for biosynthesis of macromolecules [13, 14].

lyceraldehyde-3-phosphate dehydrogenase (GAPDH) is described to be a key enzyme in the glycolytic pathway. In addition to its metabolic function, GAPDH has been implicated in various cellular processes, including DNA repair, apoptosis, and signaling [15]. One of the most important regulatory mechanisms that control GAPDH activity is redox regulation [16].

Redox regulation for example refers to the control of protein activity by the reversible oxidation and reduction of specific amino acid residues. The redox regulation of GAPDH occurs through the reversible oxidation of its three accessible cysteine residues, which can be converted to sulfenic acid (R-SOH), sulfinic acid (R-SO_2_H), or sulfonic acid (R-SO_3_H) under conditions of oxidative stress. These modifications can affect the activity of GAPDH by altering its subcellular localization, its interaction with other proteins or its enzymatic activity [17]. The reversible oxidation of GAPDH has recently been shown to be highly relevant for the triggering of the glycolysis to pentose phosphate pathway (ppp) transition [18], ensuring the supply of NADPH for glutathione disulfide (GSSG) reduction.

Here we addressed the question of whether CNP, in addition to its induction of mitochondrial dysfunction, has direct impact on GAPDH, and whether this does contribute to the selective toxicity of CNP in melanoma cells. We showed that incubation with CNP resulted in thiol oxidation of GAPDH, loss of GAPDH activity and a decrease in lactate production. The CNP induced decrease in melanoma cell viability can be mimicked with the administration of the GAPDH inhibitor heptelidic acid (HA). In addition, the application of lactate partially rescued the CNP mediated decrease in cell viability.

## 2. Materials and methods

All chemicals including cell culture medium (Dulbecco´s modified Eagle´s medium (DMEM)) were obtained from Sigma or Merck (Darmstadt, Germany) unless otherwise stated. The fetal bovine serum was from Pan Biotech (Aidenbach, Germany). The Protein Assay Kit (Bio-Rad DC, detergent compatible) was from Bio-Rad Laboratories (Feldkirchen, Germany). The enhanced chemiluminescence system (SuperSignal West Pico/Femto Maximum Sensitivity Substrate) was supplied by Pierce (Fisher Scientific, Schwerte, Germany). Penicilin/Streptomycin was obtained from Biochrom (Berlin, Germany) and Glutamax from Gibco (Darmstadt, Germany). Heptelidic Acid was purchased from Cayman Chemical Company (Distributor Biomol GmbH, Hamburg, Germany). Beta-actin antibody was purchased from Cell Signaling (Massachusetts, USA). DMSO was obtained from Roth (Karlsruhe, Germany). Horseradish peroxidase (HRP) conjugated goat anti-rabbit IgG from Dianova (Hamburg, Germany) and HRP conjugated rabbit anti-mouse IgG from Dako (Glostrup, Denmark) were used as a secondary antibody. The Sulfo Biotics Protein Redox Monitoring Kit was delivered by Dojindo EU GmbH, Munich, Germany.

### 2.1 Cell culture

The human malignant melanoma cell line A375, originally derived from a 54-year-old woman, was purchased from ATCC, Virginia, USA (ATCC^®^ CRL-1619^™^). Normal human epidermal melanocytes (NHEM), originally derived from the epidermis of juvenile foreskin or adult skin, were purchased from PromoCell (Heidelberg, Germany) (C-12400). A375 were cultured in low glucose DMEM (Sigma, Darmstadt, Germany) supplemented with 10% FBS (FBS Premium, Pan Biotech). For treatment with CNP, cells were cultured in serum-free high glucose DMEM (Sigma). NHEM were cultured in melanocyte growth medium (C-24010) with Supplementmix C-39415 (PromoCell/Bio-Connect, Huissen, Netherlands). For treatment with CNP cells were cultured in melanocyte growth medium (PromoCell/Bio-Connect, Huissen, Netherlands). Both melanoma cells and melanocytes were counted before seeding, using a Neubauer counting chamber (VWR, Darmstadt, Germany). They were grown over night to subconfluence (about 80% confluence) prior to use.

### 2.2 Cerium oxide nanoparticles

Ce IV nanoparticles (CeO_2_, 1.5 mg/ml) were purchased from Sciventions (Toronto, Canada). The nanoparticles are stabilized in sodium polyacrylate (1.27 mg/ml) and had a diameter of 1–10 nM [6].

### 2.3 Cell viability assay (MTT)

The cytotoxic effects of H_2_O_2_, CNP and heptelidic (HA) acid were measured by the MTT (3-(4,5-dimethylthiazol-2-yl)-2,5-diphenyltetrazolium bromide) assay [19]. In some experiments, lactate was added to the cells immediately prior to CNP treatment. The activity of mitochondrial dehydrogenase, as indicator of cellular viability, results in formation of a purple formazan dye. Briefly, MTT solution (0.5 mg/ml) was added to monolayer cell cultures treated for various times with the indicated substances. The cells were incubated for additional 30 minutes. The medium was removed and the cells were lysed in dimethyl sulfoxide. The formazan formation was measured at 570 nm with a FLUOstar OPTIMA plate reader (BMG Labtech, Ortenberg, Germany). The results were presented as percentage of mock-treated control which was set to 100%.

### 2.4 SDS-PAGE and Western blotting

SDS-PAGE was performed according to the standard protocols published elsewhere [20] with minor modifications. Briefly, cells were lysed after incubation in 1% SDS with 1:1000 protease inhibitior cocktail (Sigma, Taufenkirchen, Germany). After sonication, the protein concentration was determined by using a modified Lowry method (DC™ Protein Assay Kit, Bio-Rad, California, USA). 4x SDS-Page sample buffer (40% glycerol, 20% beta-mercaptoethanol, 12% SDS, 0.4% bromphenol blue) was added, and after heating the samples (20 μg total protein/lane) were applied to 12% (w/v) SDS-polyacrylamide gels. After electroblotting, immunodetection was carried out (1:1000 dilution of primary antibodies, 1:20,000 dilution of secondary antibody). Antigen-antibody complexes were visualized by an enhanced chemiluminescence system. Beta-actin was used as internal control for equal loading.

### 2.5 Visualization of redox state of thiol residues in a protein

To visualize the Redox State of thiol residues in the enzyme GAPDH the Sulfo Biotics Protein Redox State Monitoring Kit Plus was used according to the manufacturer’s protocol. Briefly, after either mock-treatment or treatment with CNP for 4 and 24 h, a so-called Protein SHifter containing a maleimide group and a DNA moiety was used to identify free thiol groups of a protein. The Protein SHifter, having a unique molecular weight of 15 kDA, binds to a free thiol group (R-SH, reduced cysteine residue), but not to the oxidized thiol group. The number of the conjugated thiol groups is directly proportional to the number of free thiols, which allows for definition of protein redox states [21]. It results in a clear mobility shift. The number of free thiol groups of a protein can be identified by SDS-PAGE. After labeling and gel electrophoresis, the SHifter moiety is cut off from the labeled protein in the gel by UV irradiation. The protein can be transferred to a PVDF membrane and detected with the corresponding antibodies.

### 2.6 GAPDH activity assay

GAPDH activity was assessed using the Colorimetric GAPDH Assay from ScienCell (Distributor Provitro AG, Berlin, Germany) according to the manufacturers protocol. The assay measures the enzyme activity in cultured cells based on the oxidization of ß-NADH to ß-NAD. GAPDH activity was determined by measuring the rate of NADH oxidation, which is proportional to the reduction in absorbance at 340 nm.

### 2.7 Measurement of intracellular lactate levels

To measure the intracellular lactate level, the L-Lactate Assay Kit from Cayman Chemicals (Distributor Biomol GmbH, Hamburg, Germany) was applied. In the assay, LDH catalyzes the oxidation of lactate to pyruvate along with the reduction of NAD to NADH. NADH reacts with the substrate to yield a final product that can be measured colorimetrically at 535 nm. Briefly, cells were treated with 300 μM CNP, 1 mM 2-DG or 500 μM H_2_O_2_ for the indicated time points (Fig 4). Thereafter, cells were harvested and centrifuged(2000xg, 10 min, 4°C). The cell pellet was used for intracellular L-lactate determination according to the manufacturer´s protocol.

### 2.8 Statistical analysis

Data were presented as mean ± standard error of the mean (SEM). Mean values were calculated from at least three independent experiments (n≥3), if not mentioned otherwise. Outliers were identified using the Grubb’s Test (significance level α = 0.05). Normal distribution of data was demonstrated via the Shapiro Wilk Test. To test data normality, the Shapiro Wilk test is the most powerful test for all types of data distribution and sample sizes (Razali and Wah, 2011). Here, the null hypothesis that the data population is normally distributed was not rejected (p > α = 0.05). Analysis of significance was performed by one-way ANOVA with post-hoc test (Dunnett test) or student’s t test (α = 0.05), as indicated, using the software Graph Pad Prism 9.1.1 (GraphPad Software, San Diego, USA). Levels of significance were defined as *p<0.05, **p<0.01 and ***p<0.001. Absolute IC_50_ values were calculated via non-linear regression (curve fit) by using the software GraphPad Prism 9.1.1.

## 3. Results

### 3.1 A375 melanoma cells were more susceptible to hydrogen peroxide and CNP than normal (healthy) melanocytes

**Fig 1:**
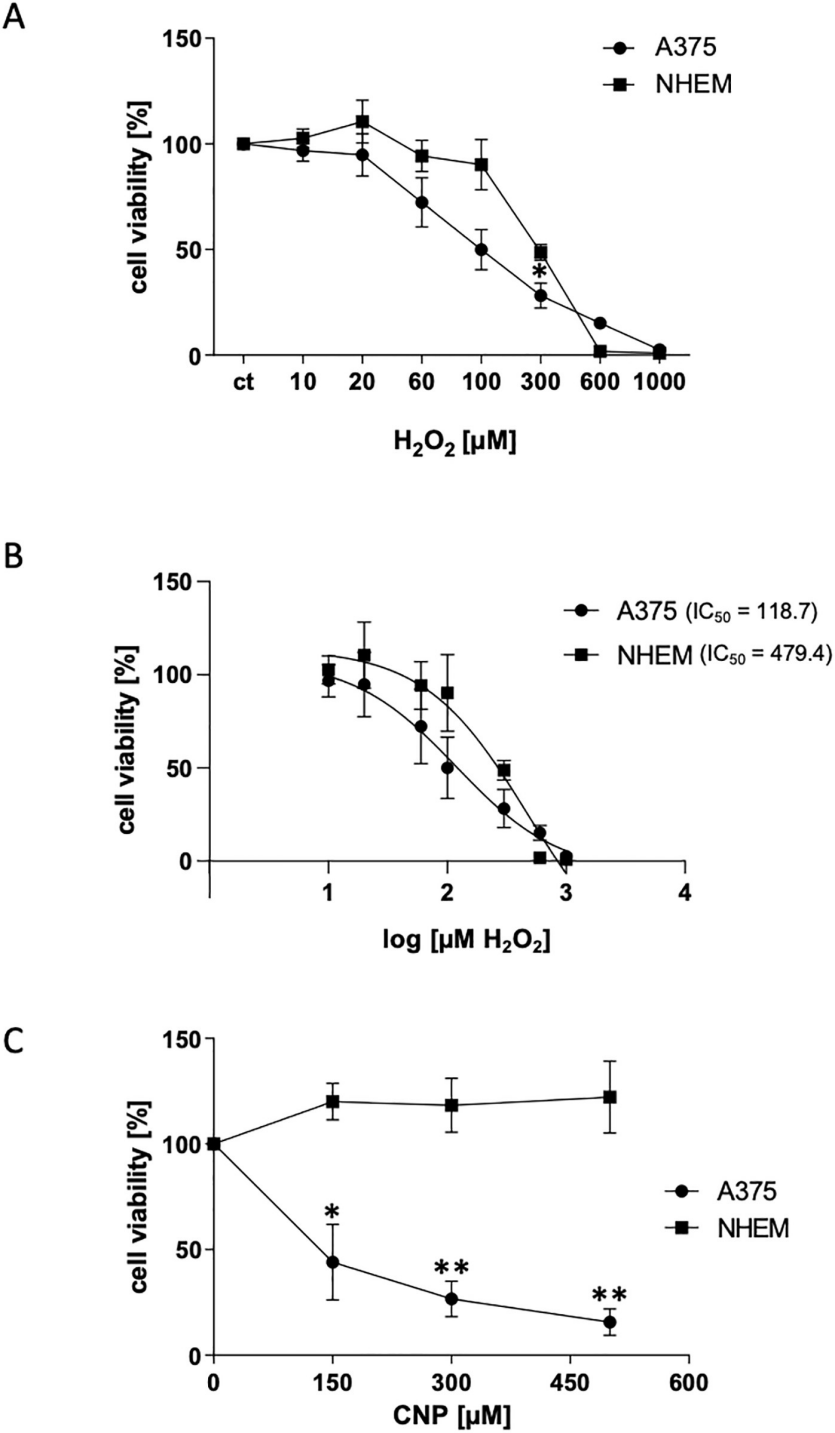
A375 melanoma cells are more susceptible to increasing concentrations of hydrogen peroxide and cerium oxide nanoparticles. (A) Subconfluent melanoma cells (A375) and normal human epidermal melanocytes (NHEM) were treated with 10 μM to 1000 μM H_2_O_2_ for 24 h. Cell viability was determined by MTT assay. The percentage of cell viability in comparison to the untreated control (set to 100%) is presented. Data are presented as means ± SEM. * p = 0.0313 A375 vs NHEM (student´s t test performed for each concentration). (B) Non-linear curve fit analysis for determination of the IC_50_ values of the data shown in (A) using Prism software (GraphPad, San Diego, USA) (A375: 95%CI, 55.81 to 275; NHEM: 95%CI, 223.6 to 1298). Data are presented as means ± SD. (C) Subconfluent A375 melanoma cells and normal human epidermal melanocytes (NHEM) were treated with 150 μM, 300 μM and 450 μM CNP for 96 h. Thereafter, cell viability was determined by MTT assay. The percentage of cell viability in comparison to the mock-treated control (set to 100%) is presented. Three independent experiments were performed (n = 3). Data are presented as means ± SEM. **p<0.01 and *p<0.05 A375 vs NHEM (student´s t test performed for each concentration).

It has been shown earlier by our group that treatment of A375 melanoma cells with CNP led to a decrease in cell viability while not affecting normal (healthy) cells [22]. An endogenous increase of the amount of reactive oxygen species such as H_2_O_2_ over a threshold which the cancer cells can no longer tolerate was identified as a possible cause for cell death [7]. To test whether the tumor cells in fact are more sensitive to exogenously added ROS because of their already existing higher base level of intracellular ROS, H_2_O_2_ at concentrations between 10–1000 μM were added to A375 melanoma cells and normal melanocytes (NHEM) for 24 h (Fig 1A). The data showed that the cytotoxic effect of H_2_O_2_ on melanoma cells already started at a concentration of >20 μM. On the contrary, NHEM tolerated higher concentrations and here the cell viability was significantly lowered at concentrations >100 μM H_2_O_2_. In summary, tumor cells reacted more sensitive on exogenously added H_2_O_2_ than NHEM, at least in a concentration range between 20–300 μM, which was substantiated by the calculated IC_50_ values by means of non-linear curve fit analysis (Fig 1B). The calculated IC_50_ value of H_2_O_2_ for A375 was 118.7 μM, whereas the IC_50_ value for NHEM was roughly four times higher (479.4 μM). At the higher H_2_O_2_ concentrations of 600 μM and 1000 μM, both cell types showed a similar cytotoxicity, which is not unusual. H_2_O_2_ is a strong oxidant, and these high concentrations are often used as positive/technical controls. Therefore, we did not include concentrations >600 μM for calculation of IC_50_ values. Furthermore, the viability of A375 and NHEM was tested after treatment with various concentrations of CNP for 96 h via MTT test (Fig 1C). Again, incubation with CNP resulted in a decrease in A375 viability while not affecting normal melanocytes. This supports earlier data [7] indicating that the SOD mimetic activity of CNP results in a further increase in intracellular H_2_O_2_ being toxic for the studied tumor cells.

### 3.2 CNP induced thiol oxidation of GAPDH

**Fig 2:**
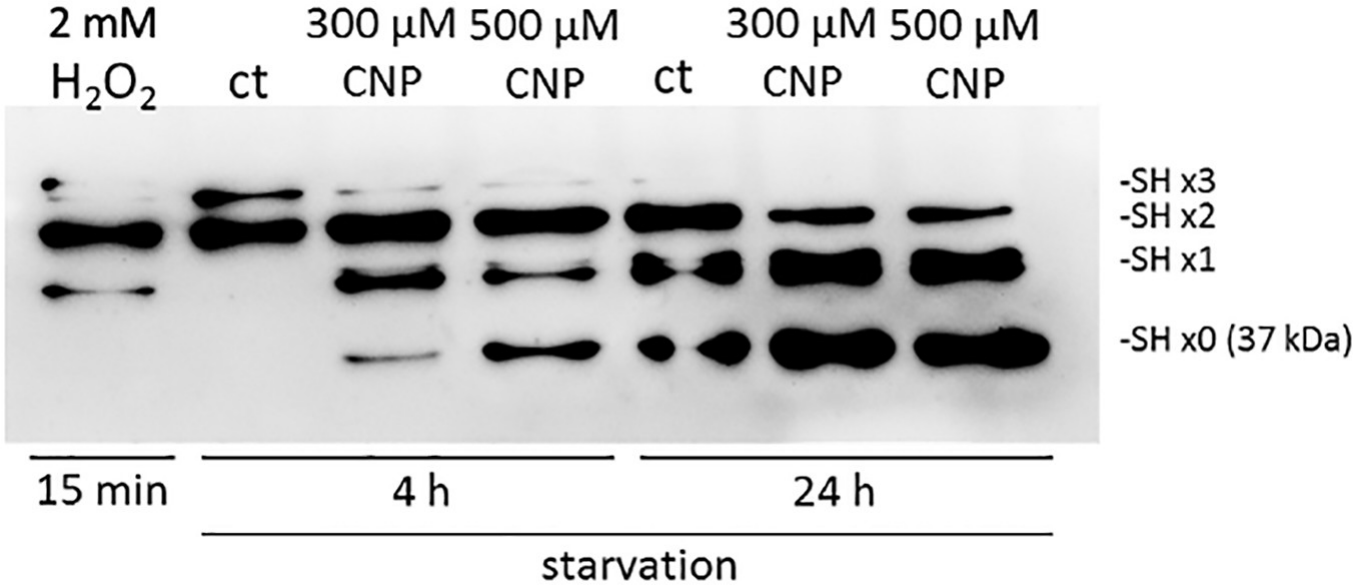
Incubation with CNP induced thiol oxidation of GAPDH in A375 melanoma cells. Subconfluent A375 melanoma cells were treated with either 2 mM H_2_O_2_ for 15 minutes or 300 μM CNP for 4 h and 24 h, respectively. Thiol oxidation was assessed via Sulfo Biotics Redox State Monitoring Kit with subsequent Western Blotting and probing for GAPDH. SHx0 to SHx3 bands refer to the amount of linker which was bound to thiol groups of the protein. If all thiol groups were oxidized prior to the incubation with the protein linker/SHifter, no SHifter was able to bind to the oxidized protein (SHx0). Experiments were performed in triplicates (n = 3).

Recently, it has been published that hydrogen peroxide is both involved in proteomic alterations and in redox signaling [23, 24], with oxidation of mostly cysteine residues resulting in functional alterations of the affected proteins [25]. As we proposed that incubation with CNP led to an increase in hydrogen peroxide, we wanted to test if CNP treatment alters thiol oxidation as H_2_O_2_ results in generation of sulfenic (R-SOH), sulfinic (R-SO_2_H) and sulfonic acids (R-SO_3_H). As cancer cells often rely on glycolysis as major energy source, we focused on thiol oxidation of the above mentioned GAPDH. To check CNP mediated thiol oxidation, we used the Sulfo Biotics Redox State Monitoring Kit (see Materials and methods). Melanoma cells were incubated with 300 and 500 μM CNP, for two different time points of 4 h and 24 h, followed by incubation with the 15kDa Protein Shifter which covalently bound to freely accessible thiol residues/reduced cysteine residues of the protein. A corresponding mobility shift was observed upon binding of this SHifter to any free thiol group (R-SH) of the target protein (Fig 2). GAPDH with a molecular weight of 37 kDa contains three accessible cysteine residues which can be successively oxidized resulting in -SHx2 and -SHx1 bands. The -SHx3 band reflects the three unoxidized thiol groups of GAPDH. However, if all thiol groups have been oxidized due to prior treatment with for example H_2_O_2_ or CNP, the binding of the SHifter is not possible, resulting in a detectable -SH x0 band. Here, the incubation with CNP resulted in thiol oxidation of GAPDH (Fig 2). In the mock-treated control (ct, 4h), the SHifter was able to bind 3 or 2 times (-SHx3 and -SHx2), indicating the unoxidized thiol groups of GAPDH. As a positive (technical) control, H_2_O_2_ was used. Incubation with 2 mM H_2_O_2_ for only 15 minutes already showed a change in oxidation with almost no detectable -SHx3 band, but with a predominant -SHx2 band and also a -SHx1 band. Treatment with either 300 μM or 500 μM CNP for 4 and 24 h resulted in an increase of thiol oxidation reflected by a strong signal of the -SHx0 band over time. This indicates that the accessible thiol groups have been oxidized prior to incubation with the SHifter. It cannot be completely excluded that the starvation process over time may result in an increase in oxidized thiols (ct, 24 h). However, treatment of the tumor cells with the SOD mimetic CNP further increased the oxidation of thiol groups compared to the controls (ct) which is reflected by the strong -SHx0 bands.

### 3.3 CNP treatment lowered GAPDH activity of melanoma cells

**Fig 3:**
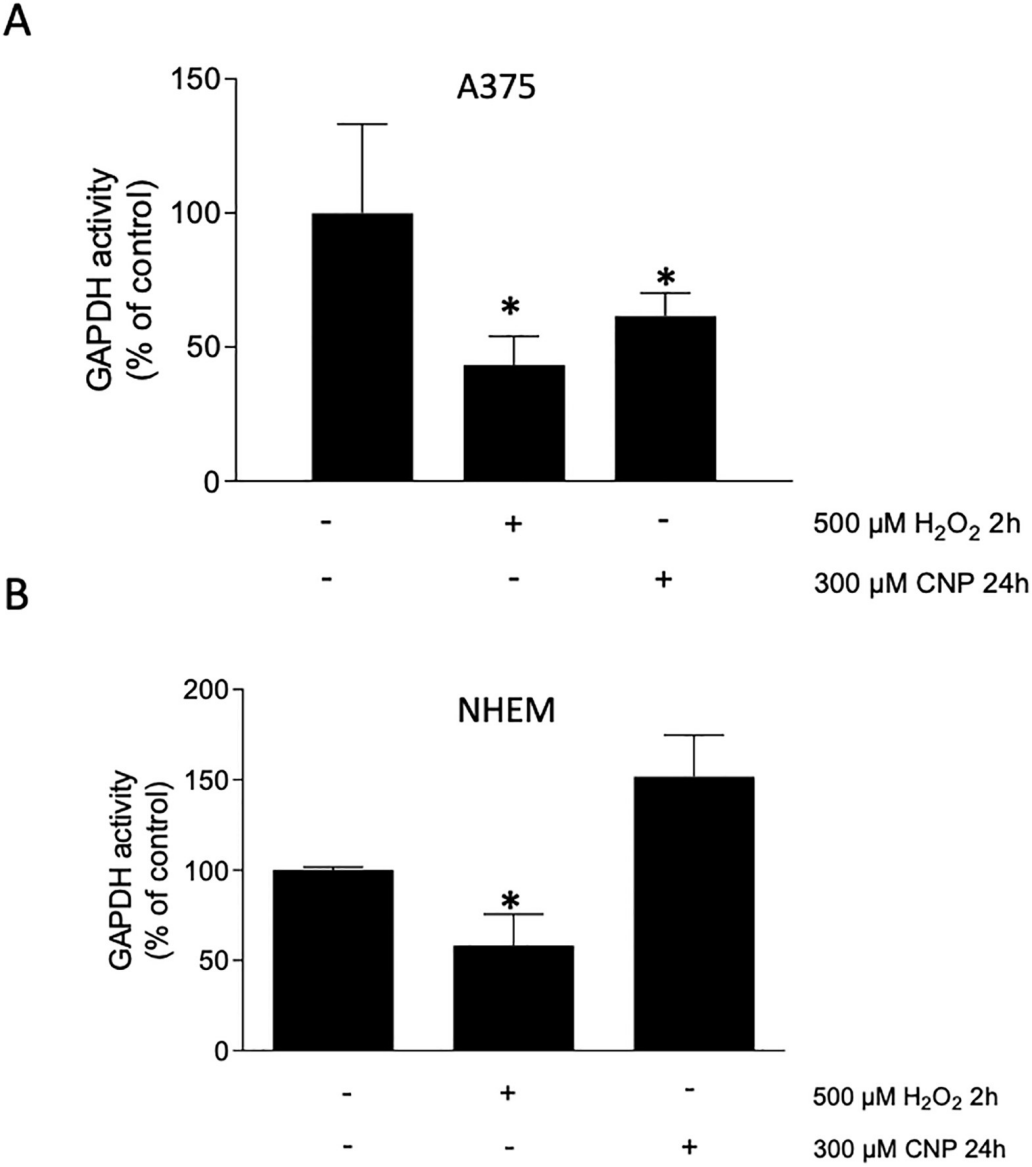
CNP lowered GAPDH activity in melanoma cells. Subconfluent A375 melanoma cells (A) or melanocytes (B) were treated with either 500 μM H_2_O_2_ for 2 h or 300 μM CNP for 24 h. The GAPDH activity was determined with the GAPDH activity Kit. Three independent experiments were performed (n = 3). The results represent means ± SEM and were normalized to cell numbers. *p<0.05 vs control (ANOVA and Dunett´s test).

We could show that CNP treatment resulted in cysteine oxidation of GAPDH (Fig 2). It has been described that thiol oxidation of GAPDH resulted in a loss in enzyme activity [26]. To study a possible decrease of GAPDH activity after CNP treatment, A375 cells (Fig 3A) and melanocytes (Fig 3B) were either incubated with CNP for 24 h or hydrogen peroxide for 2 h (control). GAPDH activity was measured using a commercially available kit. These time points were chosen as early/intermediate time points according to the time point that displayed full toxicity. Interestingly, whereas hydrogen peroxide led to a decrease in GAPDH enzyme activity in both cell lines, CNP incubation only led to a loss of activity in melanoma cells (Fig 3A) but not in the normal melanocytes (Fig 3B).

### 3.4 CNP treatment lowered lactate production in melanoma cells

**Fig 4:**
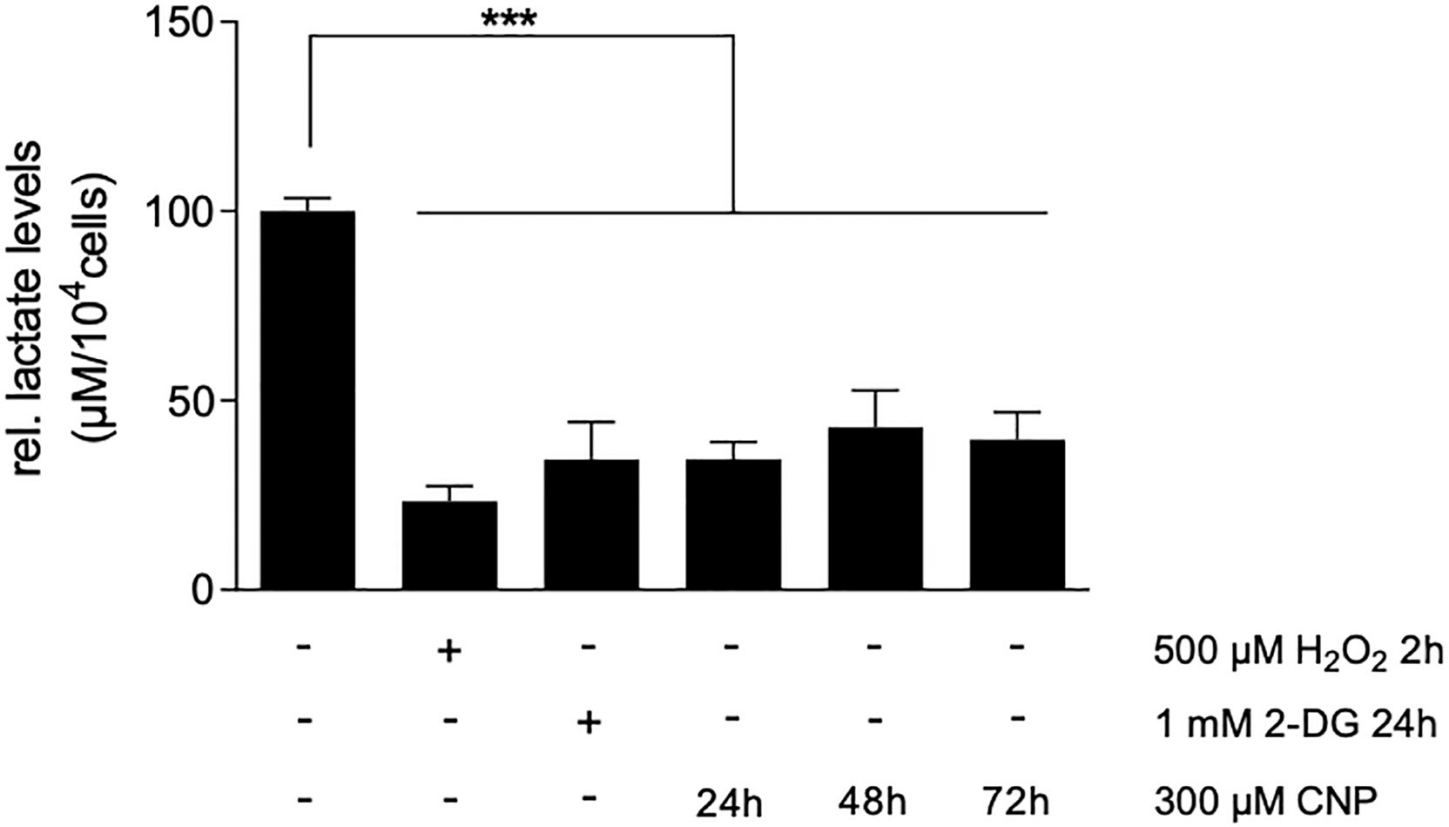
CNP lowered GAPDH activity and the intracellular lactate amount in melanoma cells. Subconfluent A375 melanoma cells were treated with 300 μM CNP for 24 h, 48 h and 72 h. As positive control, cells were treated with 500 μM H_2_O_2_ for 2 h and 1 mM 2-DG for 24 h. The L-Lactate level in melanoma cells was determined with the L-Lactate Assay Kit. Three independent experiments were performed (n = 3). The results represent means ± SEM and were normalized to the number of the cells in each sample. ***p<0.001 vs control (ANOVA and Dunett´s test).

Upon CNP mediated inhibition of GAPDH in melanoma cells, the formation of pyruvate and the subsequent reduction of pyruvate to lactate should be decreased in these cells. To test this, we measured the intracellular lactate amount after CNP treatment. A375 melanoma cells were treated with 300 μM CNP for 24, 48 and 72 hours. Hydrogen peroxide (2 h) and the glycolysis inhibitor 2-deoxy-glucose (2-DG; 24 h) served as positive controls. At all time points CNP decreased the intracellular lactate amount, which indicates a lowered glycolytic rate (Fig 4).

### 3.5 Melanoma cells are more sensitive to GAPDH inhibition than melanocytes

**Fig 5:**
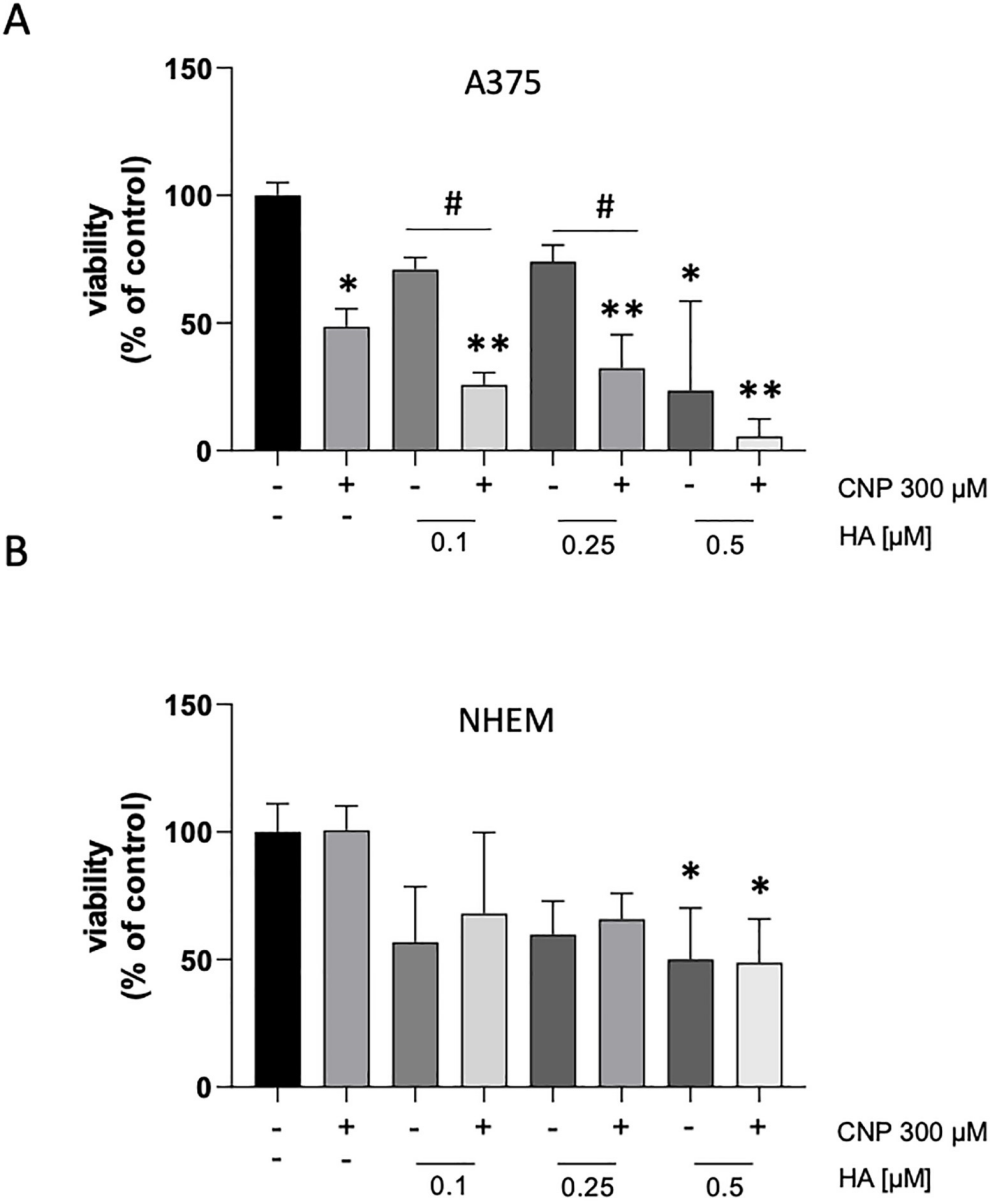
A375 melanoma cells show a higher sensitivity to GAPDH inhibition than normal melanocytes. Subconfluent A375 melanoma cells (A) or melanocytes (B) were either mock treated or treated with the indicated concentrations of heptelidic acid (HA) for 96 h. Cell viability was determined by MTT assay. The percentage of viable cells was calculated compared to the mock-treated control which was set to 100%. Three independent experiments were performed (n = 3). Data are presented as means ± SEM. **p<0.01 and *p<0.05 vs control (ANOVA and Dunett´s test). ^#^ p<0.05 vs control and HA treatment.

Next, we wanted to test whether a pharmacological inhibition of GAPDH also lowers cell viability in melanoma cells (proof of principle). For this, both melanoma cells and melanocytes were incubated with various concentrations of heptelidic acid (HA) alone or co-treated with CNP. HA is a known GAPDH inhibitor which exhibited selective toxic effects on several tumor cells [27]. A375 cells and melanocytes were pre-incubated for 1 hour with different concentrations of HA from 0.1 μM—0.5 μM, and then co-treated with CNP for additional 96 h. The lower concentrations of 0.1 and 0.25 μM HA alone only diminished the viability to approx. 75%, whereas co-treatment with CNP further decreased viability. The higher concentration of 0.5 μM induced a strong decrease of cell viability (Fig 5A). In NHEM, HA (i.e., 0.1 μM) lowered the cell viability to 50% with no additional effect of CNP, but all other doses did not further decrease viability of melanocytes (Fig 5B). In summary, addition of 300 μM CNP to low doses of HA (i.e. 0.1 and 0.25 μM, respectively) increased the effect of the GAPDH inhibitor on melanoma cell viability, whereas the viability of melanocytes was unaffected by additional CNP administration. The higher toxicity of HA alone in A375 compared to melanocytes could indicate a stronger dependance on glycolysis in the tumor cells.

### 3.6 Administration of lactate partially rescues melanoma cells from CNP induced decrease of viability

**Fig 6:**
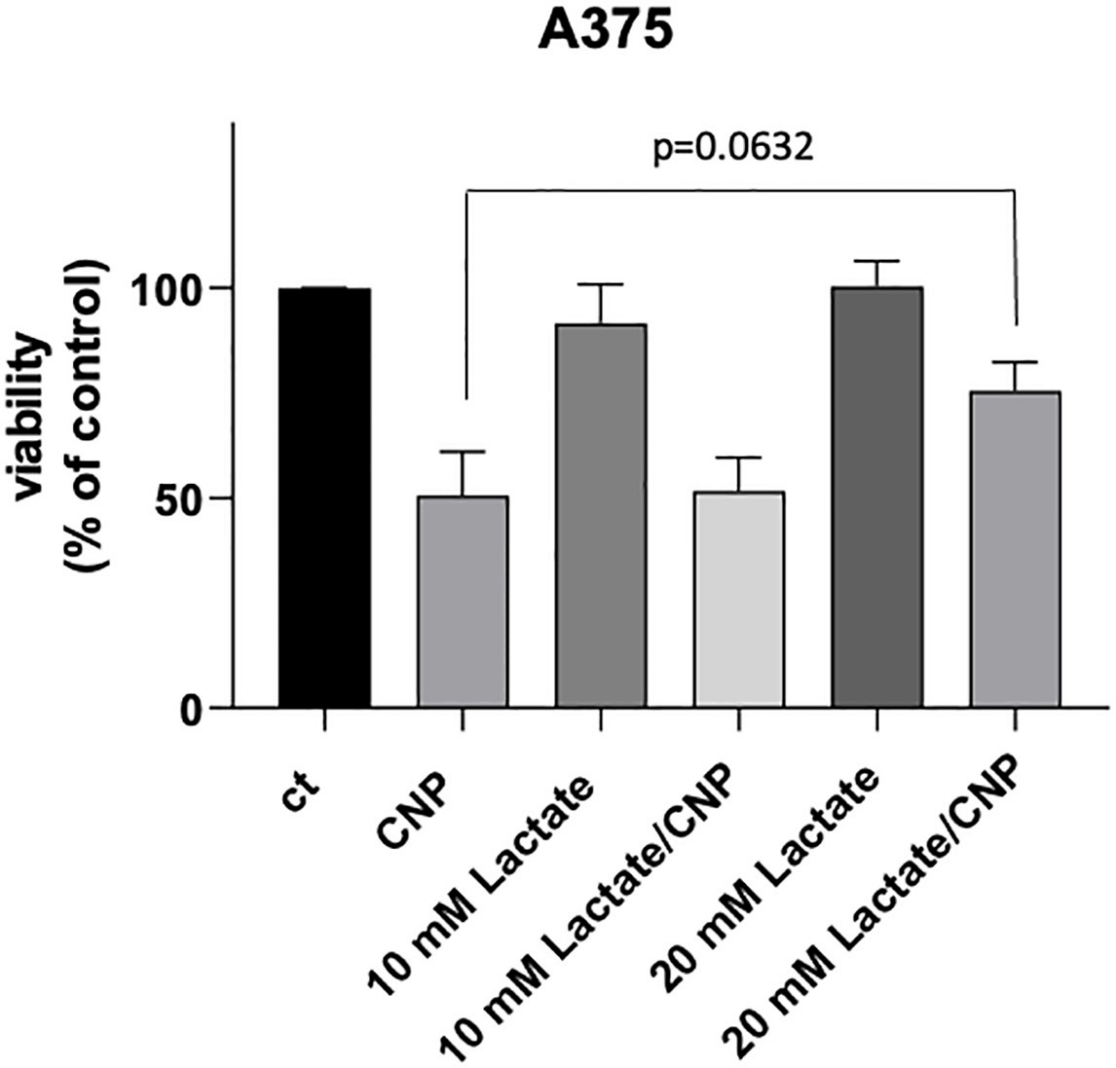
Lactate administration partially rescues CNP induced toxicity in melanoma cells. Subconfluent A375 melanoma cells were either untreated (ct) or treated for 96 h with 300 μM CNP alone, lactate (10 mM, 20 mM) alone or a combination. Cell viability was determined by MTT assay. The percentage of viable cells was calculated compared to the mock-treated control which was set to 100%. Three independent experiments were performed (n = 3). Data are presented as means ± SEM. (p value was determined by ANOVA and Dunett´s test).

Finally, to prove that the loss of viability initiated by CNP in A375 in melanoma cells is partially due to inhibition of GAPDH and subsequent insufficient energy supply, we wanted to see if supplementation with lactate rescues CNP induced toxicity (Fig 6). For this, A375 melanoma cells were incubated for 96 h with CNP alone, lactate alone or with a combination. While lactate alone had no effects on cell viability, it could be observed that the decrease in viability after 96h of incubation with 300 μM CNP was partially rescued with incubation of 20 mM lactate, but no significance could be calculated.

## 4. Discussion

Nanoparticles such as CNP are becoming more and more important in therapy, due to their versatile functions depending on size, surface properties and bioavailability [28]. Due to their mixed valance state, CNP can undergo redox reactions, which make them a promising tool in ROS related diseases and in redox regulation of enzymes involved in critical processes of cell metabolism. So far, these particles have never been shown to be toxic to normal (healthy) cells, which is another great advantage compared to standard chemotherapeutics [29, 30]. We have shown earlier that malignant melanoma cells were selectively killed by CNP based initiated formation of hydrogen peroxide [7, 31]. In this study, we showed that hydrogen peroxide selectively killed A375 melanoma cells when not exceeding a certain concentration (Fig 1) which supports the hypothesis that cancer cells are more sensitive to hydrogen peroxide (H_2_O_2_) compared to normal (healthy) cells. Increasing the basal ROS level of cancer cells to an extent that exceeds their survival strategies has been described as a novel approach in cancer therapy [32]. However, due to the rescue with PEG—catalase [7] and being aware of the glucose dependency of melanoma cells, we assumed an additional mechanism for the toxicity of CNP towards the malignant melanoma cells involving reactive oxygen species. Redox regulation by hydrogen peroxide, formed during metabolism and under oxidative stress conditions [16], has been shown to interfere with enzyme activities [33], and especially the glycolytic enzyme glyceraldehyde 3-phosphate dehydrogenase (GAPDH) is known to be one of the most prominent cellular targets of oxidative modifications regulated via thiol oxidation of accessible cysteine residues [16]. Thiol oxidation results in formation of cysteine sulfenic acid (R-SOH), which can further be oxidized to sulfinic (R-SO_2_H) or sulfonic acid (R-SO_3_H). R-SOH formation can be rescued by glutathione (GSH) [16, 34, 35]. Here we showed that incubation with CNP led to thiol oxidation of GAPDH (Fig 2), finally resulting both in a decreased GAPDH activity (Fig 3) and a decrease in intracellular lactate amount (Fig 4). This observed inhibition of GAPDH can trigger different scenarios in the cancer cells. On the one hand, inhibition of the enzyme GAPDH normally leads to a transition from glycolysis to pentose phosphate way [18], providing more NADPH necessary for the activity of the enzyme GSH reductase. This enzyme supports the cell in the defense against a rise in oxidative stress [36]. On the other hand, inhibition of GAPDH leads to inhibition of glycolysis, which may result in less ATP production. We showed that incubation of both melanoma cells and normal human epidermal melanocytes were susceptible to GAPDH inhibition by usage of the GAPDH inhibitor heptelidic acid (HA; Fig 5). However, the normal (healthy cells) remained viable, but GAPDH inhibition of A375 melanoma cells led to an almost total loss of viability. We have shown earlier that incubation with CNP led to a decrease in total ATP levels in A375 melanoma cells after 24 and 48 h of treatment [7]. This might be attributed to a decrease in ATP production via glycolysis. As A375 melanoma cells have been described to have a high glycolytic rate and a low ratio of respiration/glycolysis compared to normal cells [11]. Glycolytic activity might be crucial for these cells, resulting in cell death when glucose metabolism is impaired. To test whether an additional glucose metabolite supply, i.e. lactate as a fuel, would prevent the loss in viability, lactate was added in parallel to the incubation with CNP. Lactate accumulating in the extracellular matrix has for a long time been considered as a metabolic waste, but there is now evidence that this energy-rich metabolite can serve as a “survival” substrate for cancer cells [37]. However, albeit this lactate addition showed a tendency to rescue CNP toxicity, no statistically significant effect was observed (Fig 5). One explanation for this could be the abundance or function of monocarboxylate transporters (MCTs) [37], but this would need further clarification and was not the main focus of this study.

In this study, we showed for the first time that cerium oxide nanoparticles induced thiol oxidation of the enzyme GAPDH in A375 melanoma cells (Fig 2), resulting in loss of the enzymatic activity and a decrease in intracellular lactate formation. The fact that incubation with an GAPDH inhibitor led to a stronger decrease in viability of tumor cells than of normal (healthy) cells supports the hypothesis that melanoma cells are more prone to glycolysis than normal cells. This makes it likely that CNP exhibit their selective toxic effect on melanoma cells partially by inhibition of glycolysis via the observed thiol oxidation of the enzyme GAPDH.

## Supporting information

S1 Raw images (PDF)
