# Supplementary material for: The role of GAPDH in the selective toxicity of CNP in melanoma cells
Source: PLoS One. 2024 Mar 21;19(3):e0300718. doi: 10.1371/journal.pone.0300718 (PMC10956844; doi:10.1371/journal.pone.0300718)
Supplement: S1 Raw images — (PDF) [file pone.0300718.s001.pdf]

Western Blot antiGAPDH, 5min 34 sec exposure time:

35kDa

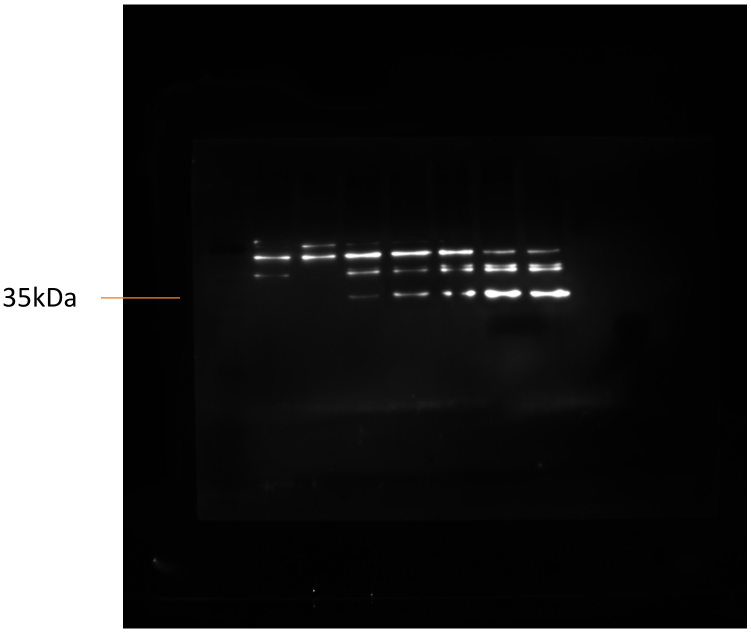

Lanes
1: H_2O_2
2: ct 4h
3: 300 µM CNP 4h
4: 500 µM CNP 4h
5: ct 24h
6: 300 µM CNP 24h
7: 500 µM CNP 24h

Membrane with prestained Marker (PageRuler):

70kDa
35kDa

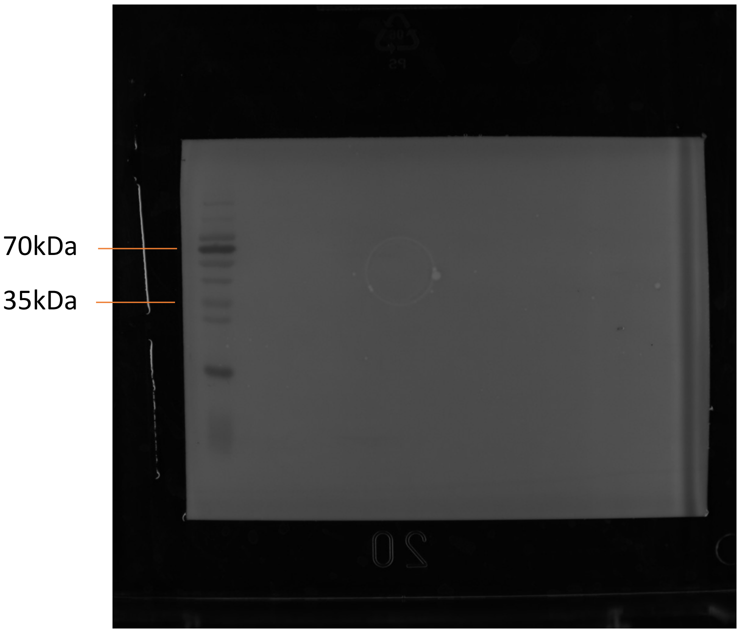

GAPDH has a molecular weight of 36kDa
